# Development of metastatic poorly differentiated thyroid cancer from a sub-centimeter papillary thyroid carcinoma in a young patient with a germline *MET* mutation – association or random chance?

**DOI:** 10.1186/s13044-021-00110-4

**Published:** 2021-08-14

**Authors:** Klara Johansson, Adam Stenman, Johan O. Paulsson, Na Wang, Catharina Ihre-Lundgren, Jan Zedenius, C. Christofer Juhlin

**Affiliations:** 1 Department of Oncology-Pathology, Karolinska Institutet, BioClinicum J6:20, Visionsgatan 4, SE-17164 Solna, Sweden; 2 Department of Breast, Endocrine Tumors and Sarcoma, Karolinska University Hospital, Stockholm, Sweden; 3 Department of Molecular Medicine and Surgery, Karolinska Institutet, Stockholm, Sweden; 4 Department of Medicine, Huddinge, Karolinska Institutet, Stockholm, Sweden; 5 Department of Pathology and Cytology, Karolinska University Hospital, Stockholm, Sweden

**Keywords:** *MET* mutation, Papillary thyroid cancer, *MET* overexpression, Poorly differentatied thyroid carcinoma

## Abstract

**Background:**

Thyroid cancer dedifferentiation is an unusual observation among young patients and is poorly understood, although a recent correlation to *DICER1* gene mutations has been proposed.

**Case presentation:**

A 28-year old patient presented with a sub-centimeter cytology-verified primary papillary thyroid carcinoma (PTC) and a synchronous lateral lymph node metastasis. Following surgery, histopathology confirmed a 9 mm oxyphilic PTC and a synchronous metastasis of poorly differentiated thyroid carcinoma (PDTC). Extensive molecular examinations of both lesions revealed wildtype *DICER1* sequences, but identified a somatic *ETV6-NTRK3* gene fusion and a *MET* germline variant (c.1076G > A, p.Arg359Gln). *MET* is an established oncogene known to be overexpressed in thyroid cancer, and this specific alteration was not reported as a single nucleotide polymorphism (SNP), suggestive of a mutation. Both the primary PTC and the metastatic PDTC displayed strong MET immunoreactivity. A validation cohort of 50 PTCs from young patients were analyzed using quantitative real-time PCR, revealing significantly higher *MET* gene expression in tumors than normal thyroid controls, a finding which was particularly pronounced in *BRAF* V600E mutated cases. No additional tumors apart from the index case harbored the p.Arg359Gln *MET* mutation. Transfecting PTC cell lines MDA-T32 and MDA-T41 with a p.Arg359Gln *MET* plasmid construct revealed no obvious effects on cellular migratory or invasive properties, whereas overexpression of wildtype *MET* stimulated invasion.

**Conclusions:**

The question of whether the observed *MET* mutation in any way influenced the dedifferentiation of a primary PTC into a PDTC metastasis remains to be established. Moreover, our data corroborate earlier studies, indicating that *MET* is aberrantly expressed in PTC and may influence the invasive behavior of these tumors.

**Supplementary Information:**

The online version contains supplementary material available at 10.1186/s13044-021-00110-4.

## Background

Papillary thyroid carcinoma (PTC) is the most common subtype of thyroid cancer, accounting for approximately 70% of cases [1]. Although subsets of PTC patients exhibit high morbidity, the disease-specific mortality is low due to the combined efficacy of surgery and adjuvant radioactive iodine (RAI) treatment [2, 3]. In terms of clinical characteristics known to increase the risk of recurrence, higher patient age, certain histological subtypes, increased tumor size as well as the presence of extrathyroidal extension, vascular invasion, and/or lymph node involvement are all recognized factors [1, 4, 5]. The v-raf murine sarcoma viral oncogene homolog gene (*BRAF*) is the most common recurrently mutated gene in PTC, with activating V600E mutations present in 50–70% of adult patients [6]. This mutation is generally associated with worse clinical outcome, and this association is especially pronounced in cases with concomitant telomerase reverse transcriptase (*TERT)* promoter mutations, occurring in 10–15% of cases [7–9]. In contrast, mutations in *RAS* proto-oncogenes, phosphatidylinositol-4,5-bisphosphate 3-kinase catalytic subunit alpha *(PIK3CA)* and AKT serine/threonine kinase 1 (*AKT1*) are generally observed in clinically less worrying PTCs [6]. In pediatric and adolescent patients, gene fusion events between various donor genes and the rearranged during transfection (*RET*) or neurotrophic receptor tyrosine kinase 1, 2 and 3 (*NTRK1*, *2* and *3*) genes are dominant [10–12].

If well-differentiated thyroid cancers dedifferentiate into poorly differentiated thyroid carcinoma (PDTC), the clinical prognosis is much worse as current medical treatments exhibit reduced effectiveness [13]. Histologically, the current World Health Organization classification recommends the Turin consensus criteria for PDTC, in which tumors display solid, trabecular or insular growth patterns, elevated mitotic counts and/or tumor necrosis as well as an absence of PTC associated nuclear changes [1]. The dedifferentiation tends to occur in old patients with large PTCs carrying mutations in *BRAF*, *TP53* and the *TERT* promoter, and several studies support the fact that an accumulation of additional mutations are possible contributors to the dedifferentiation process. In pediatric and adolescent patients however, the occurrence of PDTC is an exceedingly rare event which seems intimately coupled to mutations in the micro-RNA processor *DICER1*, a tumor suppressor gene also implicated in the development of follicular thyroid carcinoma [14, 15].

The *MET* gene encodes the MET tyrosine kinase receptor belonging to the superfamily of heterodimeric receptor tyrosine kinases (RTKs) [16]. The MET ligand, the hepatocyte growth factor (HGF), binds to MET and induces activation of the MAPK, PI3K-AKT and nuclear factor-κB pathways [17, 18]. Activation of the MET-HGF axis is firmly controlled in normal tissues since the cascade stimulates cell proliferation, mitosis, cellular motility and apoptosis. Activating *MET* mutations and gene amplifications are present in a number of malignant tumors, including sporadic and hereditary papillary renal cell carcinoma and colorectal carcinoma [19–21]. In thyroid cancer, *MET* gene mutations and fusions have also been reported, including PTCs [12, 22]. Moreover, a rare family with *RET* wildtype familial medullary thyroid carcinoma was recently shown to carry a germline, potentially causative p.Arg417Gln *MET* mutation [23].

## Case presentation

We describe a 28-year old female (denoted herein as the “index patient”) without previous medical history or family history of thyroid cancer, nor any history suggesting previous radiation exposure. She recently underwent surgery at our department for a cytology-verified sub-centimeter primary PTC with a lateral lymph node metastasis. Histopathology was consistent with a 9 mm primary PTC, but the lymph node metastasis exhibited a predominant morphology suggesting PDTC, as well as a peripheral rim with PTC morphology near the lymph node capsule. Two aspects of this clinical presentation are highly unusually encountered in routine practice: A) young patients often develop PTCs with lymph node metastases, but exceedingly seldom do we encounter dedifferentiation to PDTC in patients < 60 years of age; and B) as the PDTC was observed in the lateral lymph node metastasis adjacent to a remaining rim of PTC, this strongly suggests that the PDTC developed in the metastasis and not in the thyroid gland per se. Therefore, any underlying genetic mechanism leading to dedifferentiation would possibly be identified by parallel sequencing and comparison of data from the primary and metastatic lesion.

### Tumor attributes

**Fig. 1:**
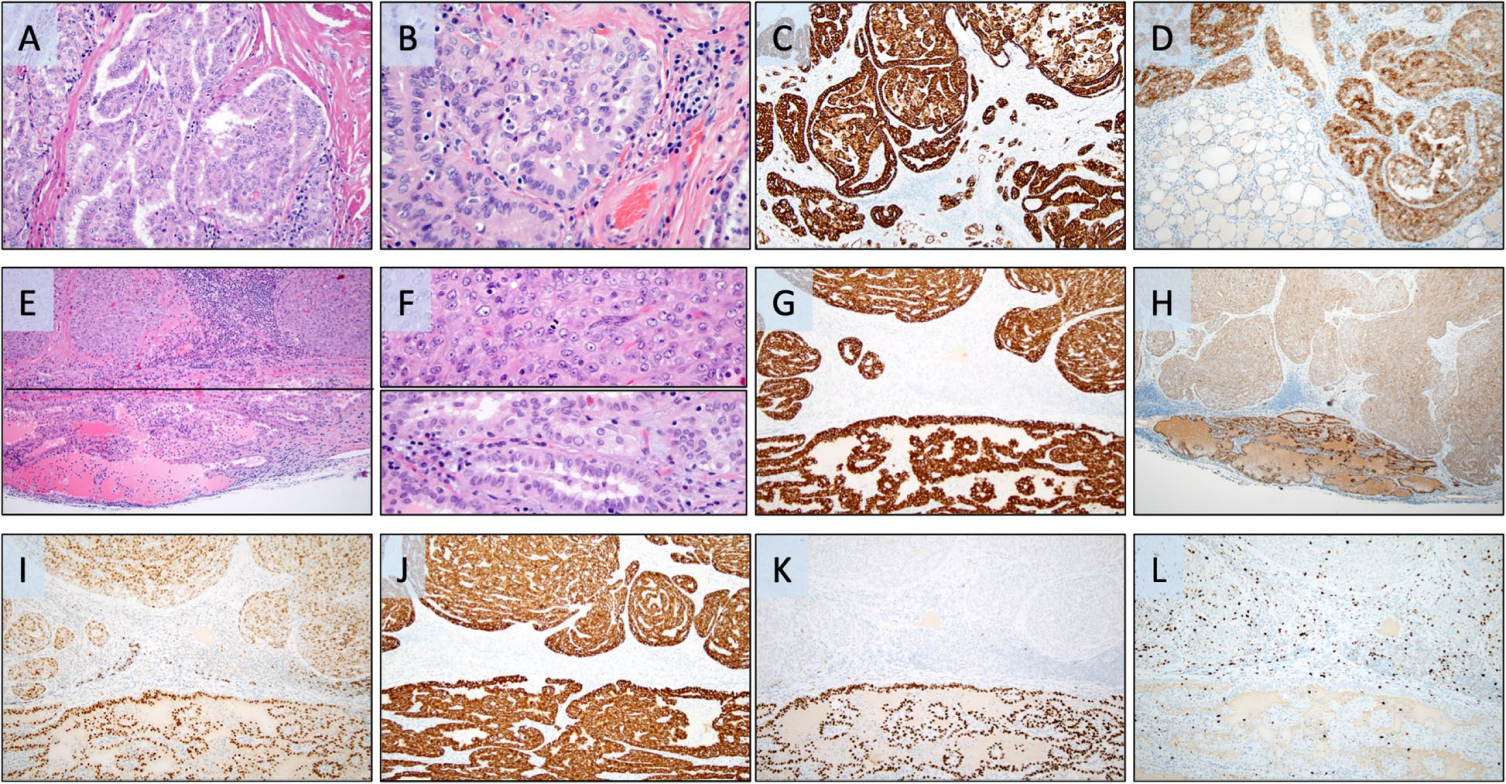
Photomicrographs demonstrating histological and immunohistochemical attributes of the primary and metastatic lesions. Magnification set to × 100 if not otherwise specified. **A** Routine hematoxylin–eosin (H&E) stain at × 200 magnification depicting the primary papillary thyroid carcinoma built-up by tumor cells with a predominantly papillary architecture. **B** Same tumor at × 400 magnification, displaying the classical nuclear features of PTC; nuclear elongation and crowding, chromatin clearing and nuclear membrane aberrancies (nuclear folds, pseudoinclusions). **C** Widespread CK19 immunoreactivity was noted within the primary tumor. **D** Strong and diffuse MET expression in the primary PTC (top) with negative staining in the normal thyroid parenchyma (bottom). **E** Routine H&E section of the lymph node metastasis with a horizontal line for clarity, a conventional PTC metastasis is visible (bottom) adjacent to an area exhibiting features of poorly differentiated thyroid carcinoma (PDTC, top). **F** Two inserts at × 400 magnification; bottom row depicts the metastatic PTC with its associated nuclear hallmarks, while the top row highlights key findings of the PDTC component, compact growth pattern, mitoses and absence of PTC-associated nuclear features. **G**-**L** Immunohistochemical analyses comparing expression in the metastatic PTC (bottom) with the metastatic PDTC (top) using the following markers; CK19 (**G**), MET (**H**), PAX8 (**I**), CK7 (**J**), TTF1 (**K**) and Ki-67 (**L**). Note how both metastatic components exhibit strong MET immunoreactivity

The histological attributes of the index patient’s tumors are illustrated in Fig. 1. DNA and RNA extraction was performed from formalin-fixated paraffin embedded (FFPE) tissues from the primary PTC as well as from the lateral lymph node metastasis using standardized methodology and protocols used in clinical routine at the Department of Pathology and Cytology, Karolinska University Hospital, Stockholm, Sweden. In brief, Maxwell 16 FFPE LEV RNA and Tissue LEV DNA Purification Kits (Promega, Madison, WI, USA) were used for nucleic acid extractions, and targeted next generation sequencing (NGS) of the tumor DNA from both the primary and metastatic lesions was performed using the Ion Torrent S5 Ion Chef (Thermo-Fisher Scientific, Waltham, MA, USA). Firstly, we sequenced DNA extracted from the primary PTC and metastatic PDTC using a small NGS panel validated for clinical routine practice (Oncomine Solid Tumor Panel, Thermo-Fisher Scientific) screening for > 1,800 mutations in 22 cancer-related genes. We thereafter interrogated tumor DNA and RNA from the same two lesions using the more comprehensive Oncomine Childhood Cancer Research Assay (Thermo-Fisher Scientific), screening for mutations in 126 genes, copy number alterations of 24 genes and > 1700 gene fusion variants in 88 genes.

### *MET* gene variant predictions

The Exome Variant Server (https://evs.gs.washington.edu/EVS/) and the Database of Single Nucleotide Polymorphisms (dbSNP; https://www.ncbi.nlm.nih.gov/snp/) were used in order to verify the minor allele frequency of the index patient’s *MET* variant. PolyPhen2 (http://genetics.bwh.harvard.edu/pph2/), “Sorting Intolerant From Tolerant” (SIFT; https://sift.bii.a-star.edu.sg/) and MutationAssessor (http://mutationassessor.org/r3/) were employed to examine if the variant had an impact on the MET protein function. The roles of this specific variant in the somatic setting were analyzed using the Catalogue of Somatic Mutations in Cancer (COSMIC) database (https://cancer.sanger.ac.uk/cosmic).

### Immunohistochemistry

MET immunohistochemistry was performed for the primary and metastatic tumor tissue using a monoclonal MET antibody (clone SP44, Ventana/Roche, Basel, Switzerland, article no: 05571219001), and standardized protocols used the Ventana automated methodology in clinical routine practice at the Department of Pathology and Cytology, Karolinska University Hospital. The staining was assessed by an experienced endocrine pathologist using light microscopy, in which the level of immunoreactivity and sub-cellular localization was scored for both tumor samples.

### PTC validation cohort

We have previously collected fresh-frozen tissues from PTC specimen from 93 selected patients operated at the Karolinska University Hospital between 1987–2005 for inclusion in unrelated projects. For this study, the 50 youngest patients were selected due to the index patient’s young age. These 50 cases were between 15–53 years old at the time of surgery and thus younger than the AJCC staging cutoff age (< 55 years). The *BRAF* and *TERT* promoter mutational status of these cases have been previously published [24]. In order to detect possible associations between PTC cases in terms of *MET* gene output, clinical parameters were retrieved, including age at diagnosis, date of diagnosis, gender, tumor size, eventual lymph node metastases, eventual distant metastases, *BRAF* V600E mutation status and *TERT* promotor mutation status, disease-free survival (endpoints: relapsed/progression or disease-free) and overall survival (endpoints: dead or alive). DNA and RNA extraction as well as cDNA synthesis was performed using the DNeasy Blood and Tissue kit (Qiagen, Hilden, Germany), miRvana miRNA isolation kit (Ambion, Austin, TX, USA) and High-Capacity cDNA Reverse Transcription Kit (Applied biosystems, Foster City, CA, United States) respectively according to the protocols provided by the manufacturers and quantified using NanoDrop ND-100 (ThermoFisher, Waltham, MA, USA).

### PCR and Sanger sequencing of the *MET* variant

Sanger sequencing verification of the *MET* gene mutation was performed in the independent PTC cohort as well as in leukocyte DNA of the index patient. The PCR reaction was performed using Platinum™ II Hot-Start PCR Master Mix (2X) (Thermo-Fisher Scientific), following the manufacturer instructions. Primers for the specific *MET* region were constructed by using Primer-BLAST (forward primer sequence 5´-TGCTCAGACTTTTCACACAAGA-3’ and reverse primer sequence 5´- GCAGTGCTCATGATTGGGTC-3´). Amplification was accomplished by following a PCR touchdown protocol. Products were purified using Invitrogen ExoSap-IT (Invitrogen, Carlsbad, CA, USA). Sanger sequencing was performed at the KIGene core facility at Karolinska University Hospital Solna using routine procedures, in order to screen for the specific p.Arg359Gln *MET* mutation. All chromatograms were visually inspected by two of the authors.

### *MET* gene quantitative real-time PCR (qRT-PCR)

We used the 7900HT Fast Real-time PCR system (Applied Biosystems) according to the manufacturer’s instructions. cDNA from PTC samples (*n* = 50) and histopathologically verified, de-identified normal thyroid tissues (*n* = 9) were normalized to a reference gene (*GAPDH*). The following Taqman Gene Expression Assays were used, *MET* as target gene (Applied Biosystems, Hs00179845_m1) and *GAPDH* (Applied Biosystems, Hs02786624_g1) as endogenous control. All specimens, tumor as well as normal, were run in triplicate and a mean value with standard deviation was calculated for each case. The relative expression was calculated using the 2^−ΔCT^ method.

### Statistical analysis

Pearson correlation test was used to compare *MET* expression, tumor size and age at diagnosis and are presented in scatterplots. Mann–Whitney U test was used to analyze differences in *MET* expression between tumors and normal thyroid tissues. Mann–Whitney U test was also used for analyzing difference in gene expression between mutated and wildtype cases. Both Mann–Whitney U test results are presented in boxplots. Where applicable, *MET* expression was classified into two ordinal groups, “high” and “low” expression, categorized by expression values above or under the tumor median. Kaplan–Meier was used to plot and present overall and disease-free survival in patients with high and low *MET* expression, log-rank test was used to calculate statistical significance. Cox regression was used to analyze covariates that could impact overall survival. The statistical analyses were performed using IBM SPSS Statistics version 27 (SPSS Inc, Chicago, IL, USA).

### Cell lines and functional experiments

Detailed information regarding the cell lines and the functional experiments are available in a Supplementary Materials and Methods file.

## Results

### Index patient NGS analyses

When interrogating DNA from the primary PTC and metastatic PDTC using the Oncomine Solid Tumor Panel, a missense variant in exon 2 of the *MET* gene (c.1076G > A, p.Arg359Gln) was found in both the primary tumor and metastasis*.* This variant was confirmed as constitutional by Sanger sequencing of germline DNA. No somatic mutational events were noted in any gene interrogated. We thereafter submitted DNA and RNA from both tumors for extended analyses using the Oncomine Childhood Cancer Research Assay, and detected a pathognomonic *ETV6-NTRK3* gene fusion in both the primary PTC as well as in the metastatic PDTC. No other cancer-related gene mutations or fusions were noted, including the thyroid cancer related genes *BRAF*, *HRAS*, *KRAS*, *NRAS*, *TERT*, *TP53* and *DICER1*.

### *MET* gene variant predictions and COSMIC database findings

In the Exome Variant Server, the specific *MET* gene variant (c.1076G > A, p.Arg359Gln) was reported in 4 out of 12,214 exomes sequenced, and the minor allele frequency was estimated to 0.03%, well below usual cut-offs for distinction of mutations (usually 0.1–1%). Moreover, consulting dbSNP, the variant exhibited a near-identical minor allele frequency of 0.000338 = 0.03%. Moreover, using the PolyPhen2 in silico prediction software, the described variant had an impact score of 0.999, meaning that is highly probable that the variant exhibited a significant consequence for MET protein function. An additional SIFT analysis yielded similar result, with the variant predicted as “damaging” (Score 0.043, cut-off 0.05). To highlight the uncertain nature of this variant using in silico analyses, we also added data from MutationAssessor, in which the variant was predicted as having a low functional impact (functional impact score of 1.575). Moreover, the p.Arg359Gln substitution is also reported in the COSMIC database as COSV59264672. The mutation has been reported in two neuroblastomas, one paraganglioma, as well as in single cases of prostatic adenocarcinoma, pancreatic adenocarcinoma and squamous cell carcinoma of the lung.

### Immunohistochemistry

The primary PTC as well as the metastatic lesion (both the PTC rim and the extensive PDTC component) exhibited strong and diffuse MET expression, distinctly stronger than adjacent normal thyroid tissue (Fig. 1D and H).

### Clinical characteristics of the PTC validation cohort

The clinical characteristics of the PTC validation cohort (*n* = 50) are shown in Table 1. The gender distribution was 4:1 (F:M), and the mean age at surgery was 36, (range: 15–53), reflecting the selective inclusion of young patients. There were 6 (12%) cases exhibiting disease relapses, 3 (6%) who had died of disease and 2 (4%) who died of other causes. 34 cases were positive for the *BRAF* V600E mutation (64%), and a single PTC exhibited the C228T *TERT* promoter mutation (2%).

**Table 1:** Clinical characteristics of the PTC validation cohort

*Characteristics*	*Number of cases*	*Percentage (%)*
**Total number**	50	N/A
**Sex**		
Male	10	20
Female	40	80
**Age at diagnosis**		
Mean ± SD	36 ± 10.1	N/A
Range	15–53	
**Tumor size (cm)**		
Mean ± SD	2.532 ± 1.337	N/A
Range	0.3–6	
**Tumor stage**		
pT1a/b	28	56
pT2	15	30
pT3	7	14
**Mutations**		
*BRAF* V600E	34	64
*TERT* promoter	1	2
**Metastasis**		
Lymph node	32	64
Distant	3	6
**Outcome at follow-up**		
Alive, no recurrence	39	78
Alive, recurrent disease	6	12
Dead of disease	3	6
Dead of other causes	2	4

*SD* Standard deviation, *N/A* Not applicable

### Absence of the *MET* mutation in PTC cohort

All 50 PTC cases were successfully interrogated for the p.Arg359Gln *MET* mutation, and the results were compared to the reference sequence by visual inspection of all chromatograms. All 50 cases displayed wildtype sequences at this position.

### *MET* gene expression in PTC cohort

**Fig. 2:**
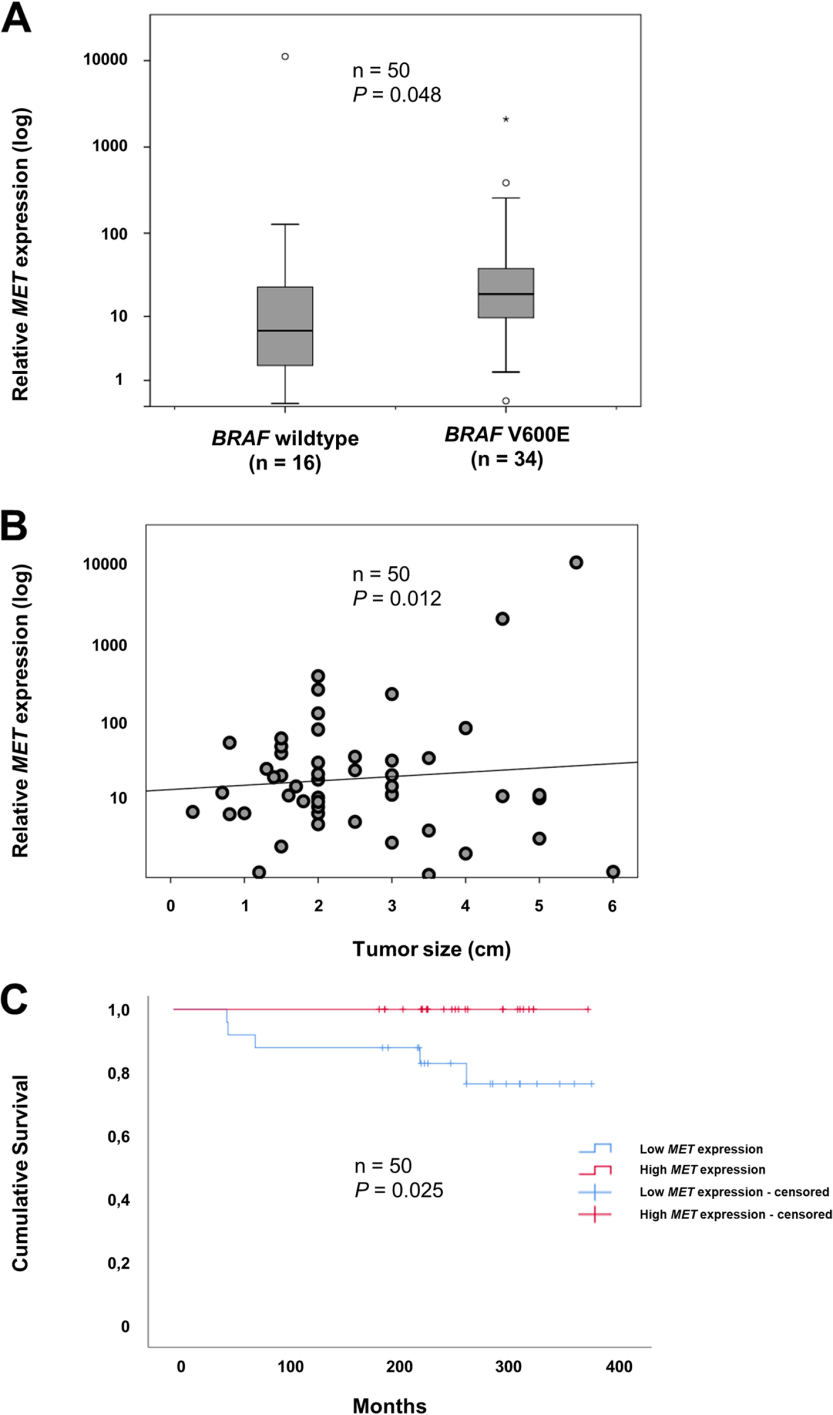
*MET* gene expression correlates to *BRAF* V600E mutation status, tumor size and overall survival in PTC. *MET* mRNA expression examined in the PTC validation cohort by qRT-PCR. **A** The cohort included 34 *BRAF* V600E mutated cases and 16 wildtype cases. The *MET* expression was significantly higher in the *BRAF* V600E mutated group compared to the wildtype group (Mann–Whitney U; *p* = 0.048). Horizontal lines represent the median *MET* expression in each group. Scale is logarithmic. **B** Increased *MET* gene expression is associated to tumor size. Scatter plot showing significant association between increased *MET* expression and greater tumor size (Pearson’s correlation test; *p* = 0.012). R^2^ Linear = 0.006. Scale is logarithmic. **C** Kaplan–Meier curve comparing overall survival in the PTC validation cohort between cases with low *MET* expression and high *MET* expression. Censored cases represent cases who were still alive or disease-free at follow up in each group

Compared to normal thyroid tissue, the *MET* expression was significantly higher in the PTC cohort (Mann–Whitney U; *p* =  < 0.001) (data not shown). *BRAF* V600E mutated cases presented with significantly higher *MET* expression than wildtype cases (Mann–Whitney U; *p* = 0.048) (Fig. 2A). There was also a significant correlation between *MET* expression and tumor size (Pearson correlation test; *R* = 0.351, *p* = 0.012) (Fig. 2B). There was no correlation to remaining parameters. Most notably from a clinical perspective, there was no significant association between *MET* expression and the presence of lymph node metastasis (Mann–Whitney U; *p* = 0.952), distant metastases (Mann–Whitney U; *p* = 0.088) or disease-free survival (Log Rank; *p* = 0.749). However, we found a significant correlation between low *MET* expression and overall survival (Log Rank; *p* = 0.025) (Fig. 2C).

### Multivariate analyses

We performed Cox regression analysis to calculate the hazard ratio (HR) for relapse and death with adjustment for covariates (Table 2). Covariates included older age (> 34.5; above the median in this cohort selected for younger cases), T stage, presence of *BRAF* V600E and *TERT* promoter mutations. For every incremental increase of T stage, the risk for recurrence increased (HR 3.569, 95% CI 1.315—9.684, *p* = 0.012), independent of other variables. Presence of a *TERT* promoter mutation was also an independent predictor of disease recurrence (HR 259.887, 95% CI 5.837—11,571.576, *p* = 0.004). T stage and *TERT* promoter mutation were also independent predictors of mortality (HR 4.423, 95%CI 0.945- 20.711, *p* = 0.009 and HR 62.818, 95%CI 1.578 -2501.380, *p* = 0.028 respectively). However, overexpression of *MET* did not predict recurrence or mortality (*p* = 0.564 and *p* = 0.964 respectively) in multivariate analyses.

**Table 2:** Hazard ratio for variables associated with relapse (A) and death (B) in PTC cohort

**Covariate**	**Univariate analyses**				**Multivariate analyses**		
	Coefficient	HR (95% CI)		*P* value	Coefficient	HR (95% CI)	*P* value
**A**							
Up-regulated *MET* expression	–0.215	0.807 (0.216–3.014)		0.749	0.453	1.573 (0.337–7.329)	0.564
Older age	–0.014	0.987 (0.263–3.703)		0.984	–0.142	0.868 (0.179–4.213)	0.860
T-stage	0.970	2.639 (1.135–6.134)		**0.024**	1.272	3.569 (1.315–9.684)	**0.012**
*BRAF* V600E mutation	–0.245	0.782 (0.195–3.135)		0.729	0.289	1.335 (0.239–7.473)	0.742
*TERT* promoter mutation	–3.871	0.021 (0.195–3.935)		**0.006**	5.560	259.887 (5.837–11,571.576)	**0.004**
**B**							
Up-regulated *MET* expression	–4.195		0.015 (0.000–20.262)	0.254	–12.212	0.000 (0.000–1.647E + 217)	0.964
Age at diagnosis	0.428		1.53 (0.256–9.191)	0.640	0.062	1.064 (0.139–8.153)	0.952
T-stage	1.133		–3.1 (0.981–9.829)	0.054	1.487	4.423 (0.945–20.711)	**0.009**
*BRAF* V600E mutation	–0.461		0.631 (0.105–3.785)	0.614	0.118	1.126 (0.101–12.498)	0.923
*TERT* promoter mutation	–3.178		23.995 (2.176–264.647)	**0.009**	4.140	62.818 (1.578–2501.380)	**0.028**

Significant *p*-values are in bold

*Abbreviations*: *HR* Hazard ratio, *CI* Confidence interval

### Plasmid transfection

Transfection of wildtype and p.Arg359Gln *MET* mutant plasmids into MDA-T32 and MDA-T41 cells revealed increased expression of pro-MET (170 kilo-Dalton, kDa) protein expression compared to mock transfected controls, as visualized by Western blot analysis. Expressional levels of mature MET (145 kDa) were similar in transfected cells and mock controls (Supplementary Figure 1). Pro-MET is the main translational product of the *MET* gene, which is then processed and modified into the MET protein which in turn localized to the cell membrane. In one of two cell lines used (MDA-T41), the mutant plasmid conferred higher pro-MET expression than the wildtype *MET* plasmid (Supplementary Figure 1).

### Wound healing assay

The migration phenotype was then explored by performing wound healing assay, which is outlined in Supplementary Figures 2 and 3. Notably, there was no obvious effect of the mutated *MET* sequence on migratory potential of the two PTC cell lines upon manual counting.

### Invasion transwell assay

Both *MET* wildtype and *MET* mutant transfected cell lines showed increased cell invasion ability comparing to mock cells (Supplementary Figure 4). However, *MET* mutant transfected cells exhibited lower capacity of invading cells compared to MET wildtype on MDA-T32 cell line.

## Discussion and conclusions

The index patient exhibited a rare manifestation of PDTC arising from a metastatic well-differentiated papillary thyroid carcinoma, driven by an *ETV6-NTRK3* fusion and possibly also influenced by a germline *MET* mutation (p.Arg359Gln). *ETV6-NTRK3* is a recurrent fusion gene event in PTC, but is to our knowledge not reported as a genetic aberrancy commonly associated to the development of PDTC in younger patients [12]. As the index tumors were negative for mutations in genes normally associated to dedifferentiation (such as *TP53*, the *TERT* promoter and *DICER1*), we therefore studied the occurrence and potential impact of the specific *MET* alteration and potential dysregulation in an extended cohort of PTCs from young patients. In the validation cohort, the majority of the patients exhibited increased tumoral *MET* expression compared to normal thyroid tissue, and there was a strong correlation to the presence of *BRAF* V600E mutations and larger tumor sizes. However, we found no additional p.Arg359Gln *MET* mutations in the same cohort. Our findings verify previous notions that up-regulation of *MET* is a common feature in PTCs, and that the index patient mutation is very uncommon, even among younger PTC patients [25–28]. Moreover, we could not see a genotype–phenotype correlation regarding p.Arg359Gln in terms of migratory or invasive potential in vitro, although overexpression of wildtype *MET* propelled the invasive behavior of the two PTC cell lines. However, given the lack of appropriate animal models, we lack in vivo evidence of this specific *MET* gene aberration actually influencing thyroid cancer dedifferentiation.

The functional consequences of the index patient’s *MET* variant is not known. A few parameters argues in favor of a true biological role: 1) the exceedingly low frequencies of this specific variant in germline DNA of the general population, 2) the pathogenic status of this variant in the majority of the performed in silico based algorithms, and 3) the known occurrence of somatic p.Arg359Gln *MET* mutations in various tumors. However, we could not see an augmented effect in the PTC cell lines transfected with mutated *MET* compared to cells transfected with a wildtype *MET* sequence only, which could imply that the variant had no clear effect on *MET* gene function. However, this assumption should also be weighed against the fact that the *MET* gene status of these *BRAF* driven cell lines were unknown, in addition to the potential influence of wildtype *MET* in these cell experiments Since *MET* is an established oncogene, a gain-of-function phenotype rather than a deleterious effect of our variant could be suspected, which is also supported by the intense MET immunoreactivity in both the primary and metastatic tumor samples in our index patient. Earlier work has shown a selective overexpression of the mutant *MET* allele in patients with germline *MET* mutations and papillary renal cell tumors, suggesting that some *MET* mutations might affect the transcriptional output, possibly through selective gene doubling [29]. Moreover, subsets of point mutations in critical regions of *MET* could most likely act as *bona fide* gain-of-function mutations [30]. Even so, since we only performed targeted NGS and not comprehensive whole-exome or whole-genome sequencing, there is always a risk that one or several unknown germline or somatic events have influenced the malignant behavior of the index tumor irrespectively of the *MET* gene variant described herein.

Interestingly, *MET* gene overexpression seems to be a recurrent event in thyroid cancer. We found that 48 (96%) PTC cases in our validation cohort exhibited increased *MET* mRNA expression compared to normal controls, although none of the cases harbored the same *MET* mutation as our index patient. Interestingly, Wasenius and co-workers did not find a significant disparity in *MET* expression between *MET* mutated and wildtype thyroid carcinomas, suggesting that *MET* gene expression is regulated in part by mutation-independent mechanisms [22]. When we compared the *MET* mRNA levels to clinical parameters in our validation cohort of PTCs, we found a significant correlation between tumor size and *MET* expression. However, unlike previous studies that suggest that increased *MET* expression is associated to a higher risk of metastases and poorer outcome, we found no such correlations [25, 26]. Nevertheless, the fact that our patients were selected based on younger ages might impact the incidence of recurrence in our cohort, as older age at surgery is strongly associated to worse outcome in thyroid cancer. Higher *MET* expression was observed in cases harboring the *BRAF* V600E mutation in contrast to wildtype cases. There is also a well-known correlation between overexpression of *MET* in patients treated with BRAF inhibitors, which is attributable to an increased *MET* signaling which reactivates the PI3K/AKT pathway and thereby circumvents the *BRAF* inhibitors [31]. The mechanism behind the observed increase in *MET* gene output in *BRAF* mutated cases without BRAF inhibitor treatment is not known.

A positive influence of invasive behavior when overexpressing *MET* in PTC cell lines was noted, but no stimulatory effects when transfecting PTC cell lines with the specific p.Arg359Gln *MET* mutation, possibly suggesting that this specific variant did not influence the metastatic properties of the PTC/PDTC in our index patient. However, we did identify an *ETV6-NTRK3* gene fusion in both the primary PTC and the metastatic PDTC. Fusion-positive thyroid carcinomas are not seldom clinically aggressive, and are predominantly PTCs, and only rarely PDTCs [32]. To our knowledge, *ETV6-NTRK3* positive PTCs dedifferentiating into PDTCs are exceedingly rare, but given the negative in vitro results of the index patient *MET* mutation, one cannot exclude that the transformation into metastatic PDTC was driven solely by the *ETV6-NTRK3* fusion in this case. Of course, the combinatory effect of the translocation and the *MET* mutation could have contributed to the dedifferentiation process, but this remains speculative.

## Supplementary Information

**Additional file 1.** Supplementary Materials and Methods.

**Additional file 2.** Western blot analysis of transfection experiments.

**Additional file 3.** Wound healing assay MDA-T32.

**Additional file 4.** Would healing assay MDA-T41.

**Additional file 5.** Transwell invasion assay.

## Data Availability

All data supporting the findings of this study are available within the article and its supplementary materials.

## Abbreviations

AKT: Protein kinase B, PK
BRAF: V-Raf murine sarcoma viral oncogene homolog B
ETV6: ETS Variant Transcription Factor 6
HGF: Hepatocyte growth factor
kDA: Kilo-Dalton
MAPK: Mitogen-activated protein kinase
MET: MET proto-oncogene, receptor tyrosine kinase
NTRK3: Neurotrophic Receptor Tyrosine Kinase 3
PDTC: Poorly differentiated thyroid carcinoma
PI3K: Phosphoinositide 3-kinase
PTC: Papillary thyroid carcinoma
RTK: Receptor tyrosine kinase
TERT: Telomerase reverse transcriptase
