# Supplementary material for: Development of metastatic poorly differentiated thyroid cancer from a sub-centimeter papillary thyroid carcinoma in a young patient with a germline MET mutation – association or random chance?
Source: Thyroid Res. 2021 Aug 14;14:19. doi: 10.1186/s13044-021-00110-4 (PMC8364030; doi:10.1186/s13044-021-00110-4)
Supplement: Supplementary file 1 — Additional file 1. Supplementary Materials and Methods. [file 13044_2021_110_MOESM1_ESM.docx]

**Supplementary Materials and Methods**

*Cell lines*

Two commercially available PTC cell lines, MDA-T32 and MDA-T41, were purchased from the American Type Culture Collection (ATCC, Manassas, VA, USA) in 2018 and have been previously used in our laboratory [23]. Cells were cultured in RPMI-1640 medium (Gibco/Thermo-Fisher Scientific) supplemented with 10% fetal bovine serum (Thermo-Fisher Scientific), 1% L glutamine (Thermo-Fisher Scientific) and 1% NEAA (Gibco/Thermo-Fisher Scientific). MDA-T41 and MDA-T32 carry a *BRAF* V600E mutation, and MDA-T32 also carries a -124 (C>T) *TERT* promoter mutation.

*Plasmid construct and transfection*

The c.1076G>A, p. Arg359Gln mutant and wildtype *MET* plasmids (both 4245 base pairs, sequences available upon request) were synthesized by Thermo-Fisher Scientific using the GeneArt service. The PTC cell lines MDA-T32 and MDA-T41 were transfected with 1 µg of wildtype and mutant *MET* plasmids using Lipofectamine LTX and PLUS reagents kit (Thermo-Fisher Scientific) and cell pellets were collected after 48 hours.

*Western blotting*

In order to validate the transfection efficacy of the *MET* plasmids, transfected cell lines were harvested and extracted for protein using RIPA Lysis and Extraction Buffer (Thermo-Fisher Scientific). 30 µg of protein was used. The membranes were incubated with a rabbit monoclonal MET primary antibody (clone D1C2, Cell Signaling Technology, Danvers, MA, USA) diluted 1:1000. A rabbit monoclonal GAPDH antibody (clone 14C10, Cell Signaling Technology) signal served as loading control.

*Wound healing assay*

Cells were seeded on 6-well plates and transfected with wildtype and *MET* mutated plasmids. Upon reaching a mono-layer, a wound was scratched through the plate center using a sterile pipette. Time-lapse microscopy was initiated directly following pipette scratching, and the wound healing area was calculated via the ImageJ software. Representative photomicrographs of cells were taken at 0h, 4h, 9h, and 11h respectively. Experiments were repeated three times.

*Invasion transwell assay*

MDA-T32 and MDA-T41 cells were seeded on BioCoat™ Matrigel® Invasion Chambers with 8.0 µm PET Membrane (Corning). Transwell inserts were pre-coated with Matrigel stock solution. Non-serum and 10% serum medium were used inside and outside chambers, respectively. After 20h incubation in a 37 °C incubator, invaded cells were fixed and stained with crystal violet. Stained cells were counted from nine different fields of view on each chamber, and the average sum of cells was calculated for each group.
