# Supplementary figures and images for: Development of metastatic poorly differentiated thyroid cancer from a sub-centimeter papillary thyroid carcinoma in a young patient with a germline MET mutation – association or random chance?
Source: Thyroid Res. 2021 Aug 14;14:19. doi: 10.1186/s13044-021-00110-4 (PMC8364030; doi:10.1186/s13044-021-00110-4)

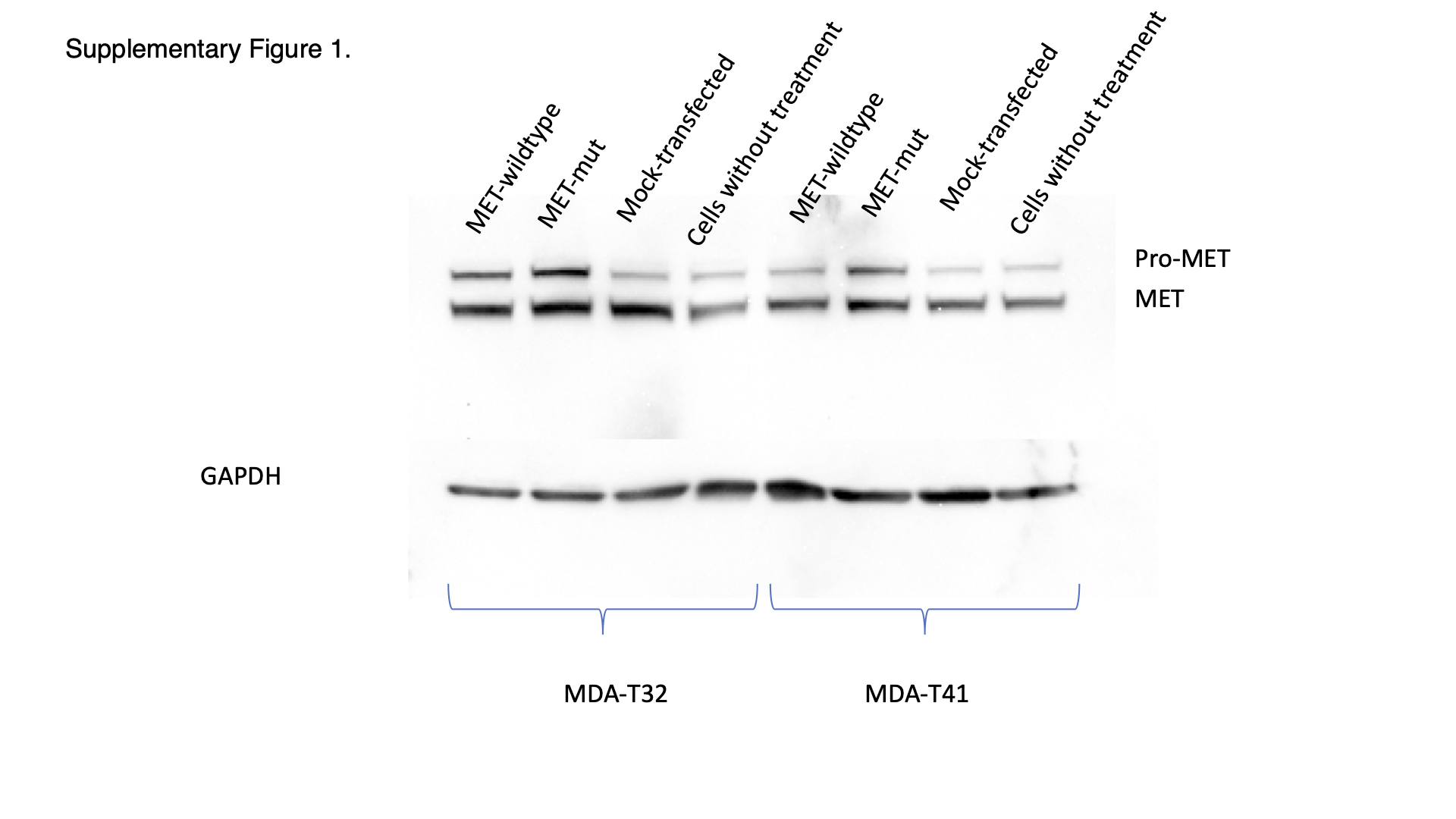

Supplement: Supplementary file 2 — Additional file 2. Western blot analysis of transfection experiments. [file 13044_2021_110_MOESM2_ESM.tiff]

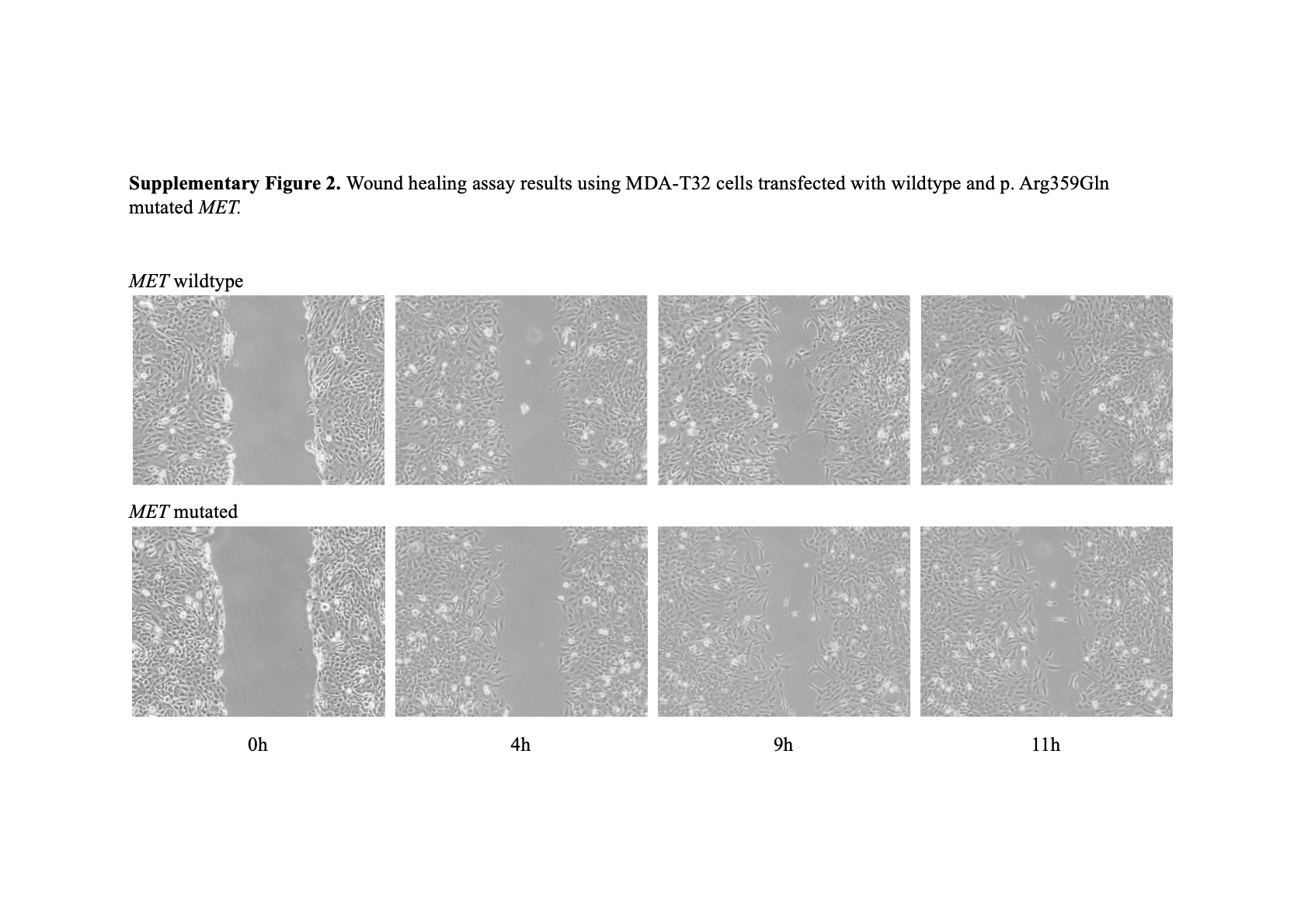

Supplement: Supplementary file 3 — Additional file 3. Wound healing assay MDA-T32. [file 13044_2021_110_MOESM3_ESM.tiff]

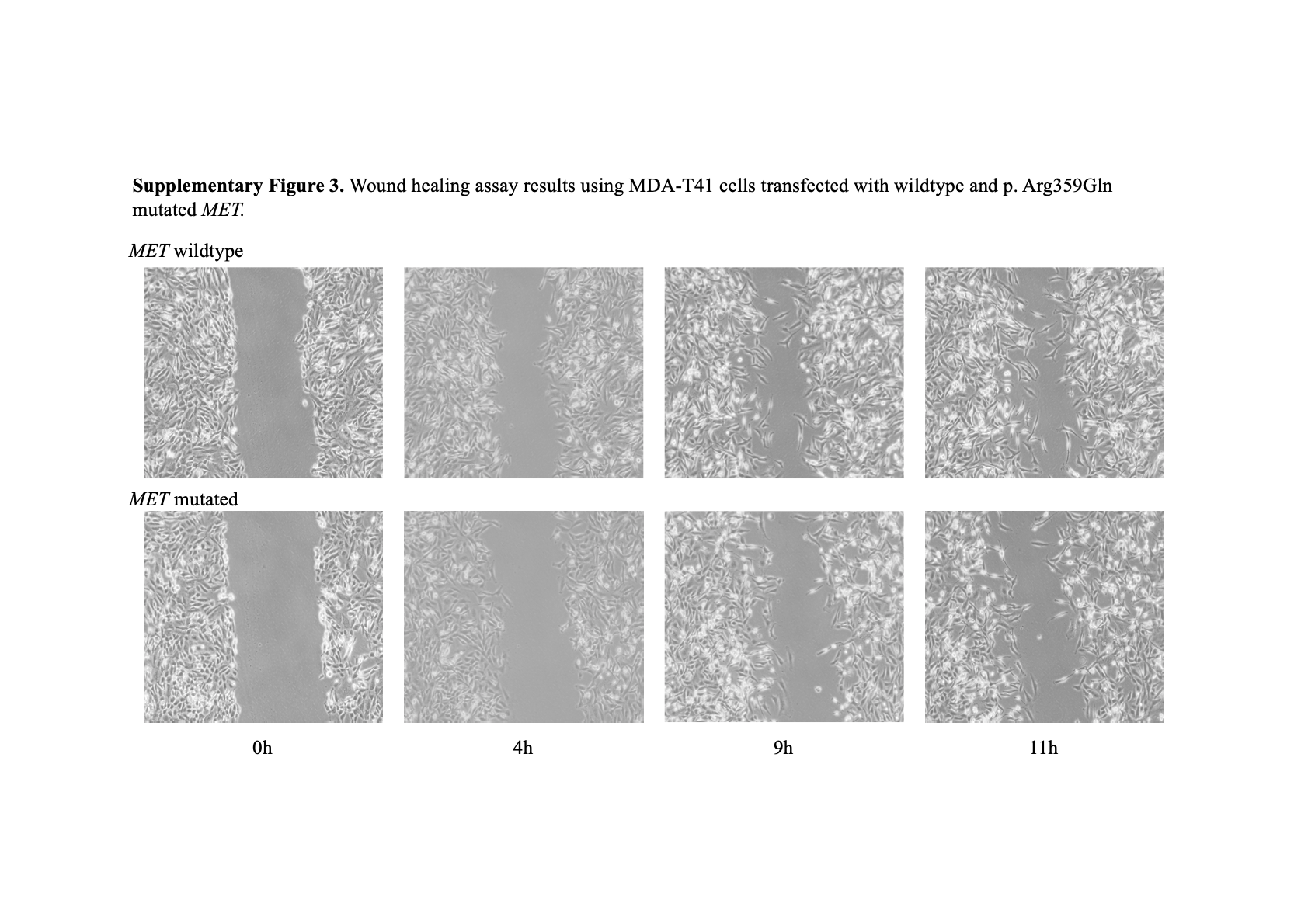

Supplement: Supplementary file 4 — Additional file 4. Would healing assay MDA-T41. [file 13044_2021_110_MOESM4_ESM.tiff]

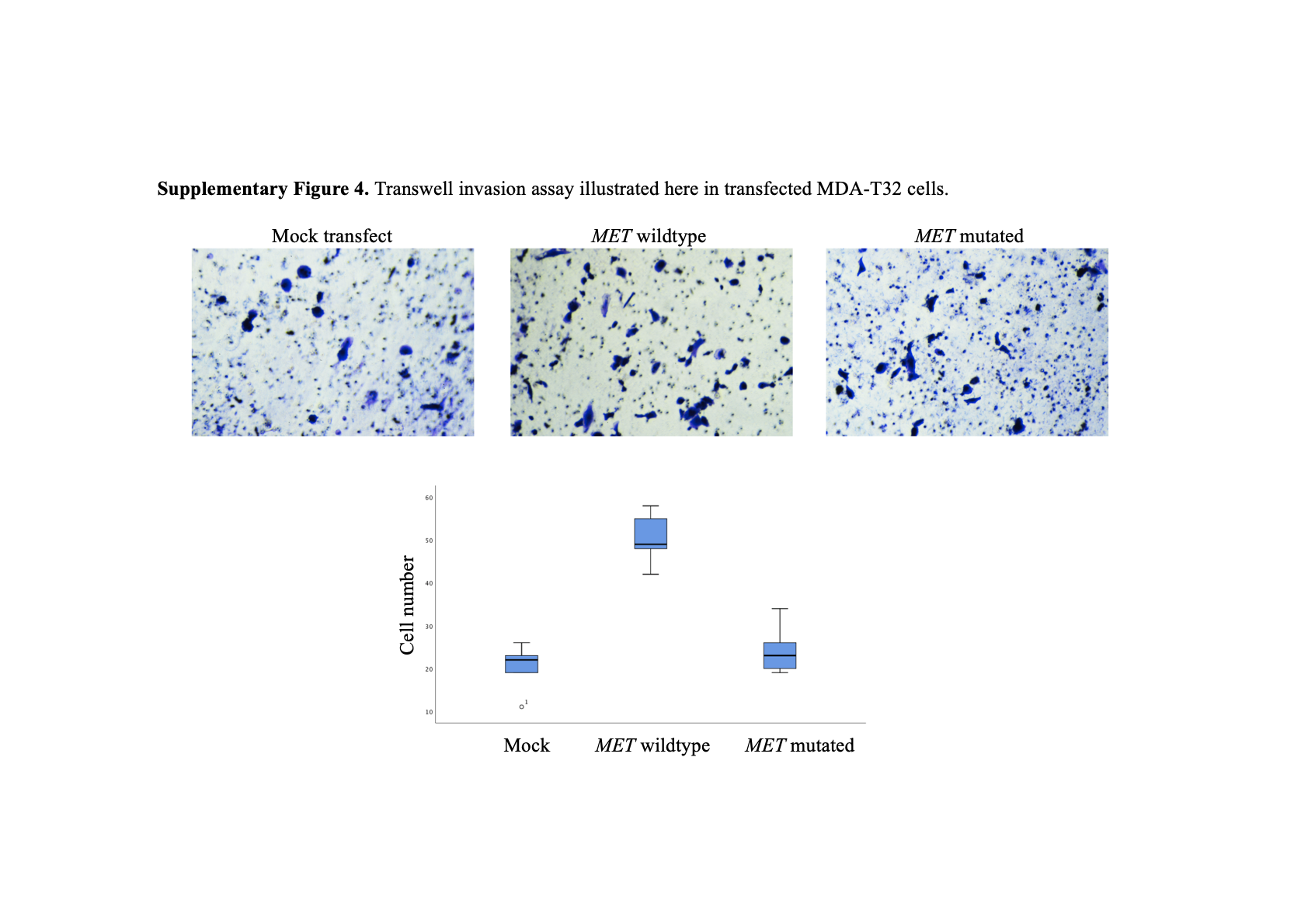

Supplement: Supplementary file 5 — Additional file 5. Transwell invasion assay. [file 13044_2021_110_MOESM5_ESM.tiff]
